# The relationship between cognition and functioning in schizophrenia: A semi-systematic review

**DOI:** 10.1016/j.scog.2021.100217

**Published:** 2021-09-29

**Authors:** Saifuddin Kharawala, Claudia Hastedt, Jana Podhorna, Hemlata Shukla, Bregt Kappelhoff, Philip D. Harvey

**Affiliations:** a Bridge Medical Consulting Ltd., 2 Marsault Court, 11 Kew Foot Road, Richmond TW9 2SS, United Kingdom; b Boehringer Ingelheim International GmbH, Binger Strasse 173, 55216 Ingelheim, Germany; c Boehringer Ingelheim bv, De Boelelaan 32, 1083 HJ Amsterdam, the Netherlands; d University of Miami Miller School of Medicine, 1120 NW 14th Street, Suite 1450, Miami, FL, United States of America

**Keywords:** Schizophrenia, Cognitive impairment, Functional capacity, Real-world functioning

## Abstract

In schizophrenia, impairments in neurocognition (NC) and social cognition (SC) are associated with reduced functional capacity (FC) and poor real-world functioning (RWF).

In this semi-systematic review, we examined this association across a range of research questions.

We conducted a systematic search in Embase and MEDLINE from 2005 to 2019, and conducted additional pragmatic searches. After screening of titles, abstracts and full-texts, we included 564 citations, of which 44 (26 primary studies, 15 systematic reviews and 3 narrative reviews) were prioritized for reporting.

Both NC and SC were significantly associated with functioning, with slightly stronger association for SC. Effect sizes were generally larger for FC than for RWF. NC showed stronger associations with occupational functioning and independent living, and SC with social functioning. Baseline cognition predicted long-term RWF up to 20 years of follow-up, though long-term data were limited for SC. Cognitive remediation improved RWF functioning, especially when it was combined with psychosocial rehabilitation.

SC mediated the relationship of NC with functioning. Negative symptoms appeared to mediate and moderate the association of cognition with functioning. Other factors involved included severity of cognitive dysfunction, metacognition, depression and choice of RWF instrument.

We discuss potential implications for studies of pharmacological cognitive interventions in schizophrenia – the relevance of both NC and SC, the advantage of adjunctive psychosocial rehabilitation, the role of relevant moderating and mediating variables, and the challenges with RWF instrument selection. Successful cognitive interventions could allow patients with schizophrenia to improve their potential for community functioning.

## 1 Introduction

Cognitive impairment is a fundamental part of schizophrenia and a key indicator of outcome. Some cognitive impairments often appear before the onset of psychosis (Carrión et al., 2018), and the decline in cognitive performance may then be sustained through the chronic stages of schizophrenia, even in middle age (Fett et al., 2019).

Most patients with schizophrenia experience a broad range of cognitive impairments, in neurocognition (NC) as well as social cognition (SC). The key domains of NC that have been studied in schizophrenia include processing speed, attention/vigilance, working memory, verbal learning and memory, visual learning and memory, reasoning and problem solving, verbal comprehension, and verbal fluency. A neurocognitive composite factor score is often calculated as a composite score across all NC domains. The key domains of SC include emotional processing or emotion perception (the ability to infer emotional information from facial expressions, vocal inflections or some combination of these), social perception and knowledge (the ability to identify social roles, societal rules, and social context), theory of mind (ToM; the ability to understand the mental states [beliefs, knowledge and intentions] of other people and infer that these may differ from one's own), and attributional bias (the process of attaching meaning to behavior, i.e. finding reasons for one's own or another's behavior; Fett et al., 2011; Irani et al., 2012; Nuechterlein et al., 2004; Pinkham et al., 2016; Pinkham et al., 2018; Ventura et al., 2009).

In patients with schizophrenia, recovery is no longer measured by an absence of psychotic symptoms; but also by functional recovery, which has become an important focus of treatment (APA, 2004; Harvey and Bellack, 2009; NICE, 2014). Despite advances in pharmacological and psychological treatments, schizophrenia remains one of the most disabling illnesses, with patients continuing to experience impairment in activities of daily living (ADL), working capacity and social functioning (Galderisi et al., 2014; Harvey et al., 2019a). Functioning in schizophrenia is generally described in terms of two distinct constructs: real-world functioning (RWF)/community functioning (“what one actually does in the real world”) and functional capacity (FC) or competence (“what one can do under optimal conditions”) [Gupta et al., 2012]. RWF is generally assessed through third-party ratings of patient behavior in real-world situations, or measurement of objective milestones such as marriage or competitively-obtained employment. In the literature, RWF is also referred to as community functioning, community living and community outcome.

The components of RWF include social functioning (social connectedness and social interactions in the community; described in terms of participation in social activities, interactions with family and friends, etc.), occupational/vocational functioning (functioning at work or school; assessed in terms of work skills, adaptive functioning with reference to work, work performance, etc.), and independent living (day-to-day functioning, including both basic-ADL [BADL] and instrumental-ADL [IADL]; assessed in terms of personal care and appearance, diet, housekeeping, self-care, finances, travel, etc.). An example of an instrument used for assessing RWF is the Specific Level of Functioning (SLOF) Scale, which measures actual real-world functioning in terms of interpersonal relationships, social acceptability, participation in activities and work skills; and can be completed by the subject or by a clinician, family member, or close friend selected by the subject (Browne et al., 2016). FC is the ability to perform tasks relevant to everyday life in a structured environment, guided by an examiner. FC is assessed through specific tasks or role-play, usually in controlled settings by asking participants to simulate real-world activities, such as holding a conversation, selecting grocery items to prepare a meal, planning a trip using public transportation, etc. Recent advances have included virtual reality simulations of everyday tasks such as shopping and travel (Keefe et al., 2016) and veridical simulations of technology-based tasks such as using a ticket kiosk or banking at an ATM (Czaja et al., 2017). Alternative terms for FC in the literature include “laboratory assessment of instrumental skills and social problem-solving ability” (Green et al., 2015), functional competence, intermediate measure of functioning, and “proxy” measure of functioning. Further, performance-based measurement of social skills, often referred to as “social competence” is a socially relevant FC measure. An example of an instrument used for assessing FC is the Social Skills Performance Assessment (SSPA), in which subjects are assessed in two role-play situations (simulated scenarios) where their social skills are evaluated (Browne et al., 2016).

The substantial literature in this area suggests that the relationship between cognition and functioning is complex, with multiple mediating and moderating factors which may affect the ability of cognitive improvements to translate into better community functioning. A detailed and holistic understanding of the inter-relationships is needed.

While a number of reviews, including several systematic literature reviews (SLRs) and meta-analyses, have focused on specific aspects of the association of cognition with functioning, we have not identified any recent review that has attempted to synthesize data across multiple facets of this association in a structured manner. Many additional studies have been published since the earlier detailed reviews on this subject (e.g., Bowie and Harvey, 2006), and an updated review of the state of the field is needed. To address this gap, we conducted a semi-systematic review of the literature to assess the association of cognition and functioning in schizophrenia across a range of research questions, and to understand how cognitive interventions may improve functioning. The following aspects were covered: 1) the overall association of cognition with functioning, including relationships across individual domains; 2) the association of cognition with change in functioning over time; 3) the effect of cognitive interventions on functioning; 4) factors influencing the association of cognition with functioning; and 5) the relationship between FC and RWF.

**Fig. 1:**
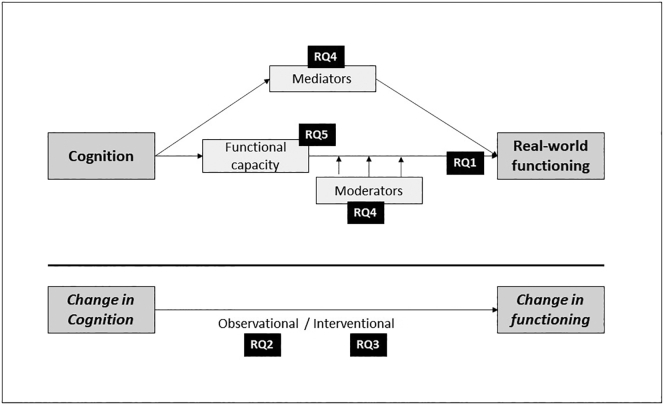
Facets of the inter-relationship of cognition with functioning. RQ, research question (refers to the research questions of interest in this review article).

Fig. 1 summarizes the facets of the relationship that are covered in this review, highlighting the research questions of interest.

## 2 Methodology

Publications were identified by a literature search, following which the highest-quality, most relevant publications were prioritized.

### 2.1 Search strategy

We conducted systematic searches on October 15, 2019 in the Embase® and MEDLINE® databases using Embase.com to identify articles published from 2005 to 2019 (details in Tables S1 and S2); combining disease facet terms including ‘schizophrenia’ and ‘cognitive impairment associated with schizophrenia’ (CIAS) with outcomes facet terms for functioning (‘functioning’, ‘activities of daily living’, ‘ADL’, ‘social interaction’ etc.) and cognition (‘cognition’, ‘cognitive function’, ‘cognitive performance’ etc.). We also conducted bibliographic searches; keyword-based pragmatic searches in PubMed, Google, and Google Scholar (including relevant literature prior to 2005); and searches in conference proceedings.

### 2.2 Study selection

A single reviewer screened citations, and a second reviewer conducted an independent quality check on the selected citations. Titles and abstracts were reviewed first, then full-text articles. Screening criteria covered patient population (patients with schizophrenia, schizoaffective disorder and psychosis), intervention (all cognitive interventions), outcome of interest (publications assessing the relationship between cognition and functioning were included, while those assessing only cognitive or only functioning outcomes were excluded), and study design (prospective and retrospective cohort studies, cross-sectional studies and registries, case-control studies, randomized controlled trials [RCTs], non-RCTs, and single-arm studies were included; case reports and case series were excluded).

### 2.3 Prioritization

To avoid duplicating information and to provide a useful overall summary, we further screened articles based on study design, sample representativeness, sample size, duration of follow-up and relevance to research questions; giving preference to well-conducted systematic reviews and large longitudinal studies with representative samples. The prioritized publications were all of high quality and adequately represented the overall literature.

### 2.4 Data extraction

A single reviewer extracted the following data: study design (e.g., prospective cohort study, systematic review etc.), population (diagnosis [schizophrenia/psychosis], sample size, follow-up duration etc.), assessment measures (e.g. MATRICS Consensus Cognitive Battery [MCCB] for cognition, Global Assessment of Functioning [GAF] for functioning etc.), and outcomes (e.g. association between cognition and function, effects of interventions, relationship between FC and RWF etc.). An independent reviewer checked data quality.

### 2.5 Quality assessment

A qualitative assessment of the strengths and weaknesses of each prioritized publication was conducted using the Newcastle–Ottawa Scale (NOS) for observational studies (Herzog et al., 2013; Wells et al., 2012) and A Measurement Tool to Assess Systematic Review (AMSTAR) for systematic reviews (Shea et al., 2009). While no studies were excluded based on the quality assessment, it informed our interpretation of the data, as well as our conclusions.

## 3 Results

### 3.1 Overview of studies

**Fig. 2:**
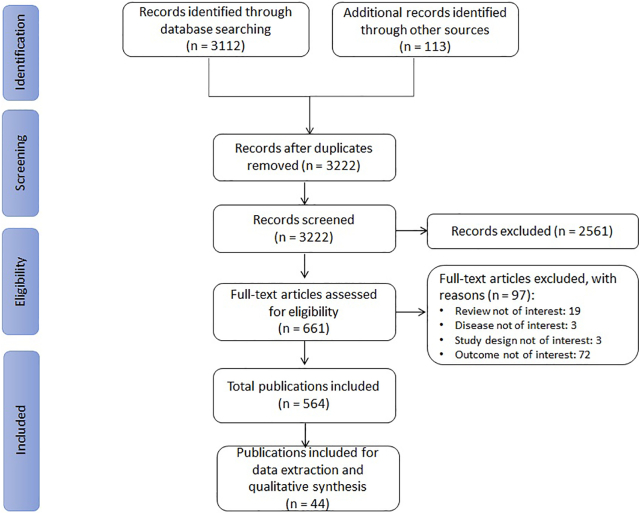
PRISMA flowchart for the identification, selection, and prioritization of articles in the review.

The Embase/MEDLINE searches retrieved 3112 citations and supplementary searches 113 citations (Fig. 2). After title/abstract and full-text screening, 564 citations were included, of which 44 were prioritized for data extraction and qualitative analysis. This included 26 primary studies (13 cross-sectional and 13 longitudinal studies) and 18 reviews (15 SLRs and 3 narrative reviews).

#### 3.1.1 Association of cognition with functioning, including relationships across individual domains

Table 1 summarizes the key publications reporting data on the association of cognition with functioning. Since the literature in this area is extensive, we mainly summarize data from review articles (3 SLRs), with a few high-quality primary studies (4 cross-sectional studies).

**Table 1:** Publications on the overall association of cognition with functioning.

Publication name [Quality]	Type of publication	Sample size/No. of studies (total sample size)	Population	Cognitive domains assessed	Functioning domains assessed	Key results
Halverson 2019 [Q = High]	SLR with meta-analysis	Total studies: 166 (*n* = 12,868)	SCZ spectrum disorders	NC and SC	Domains: social, occupational, independent living (FC and RWF)	NC (overall and individual domains) was associated with functioning, with small-to-medium^a^ ES (ES using û_p_ for overall NC and functioning was 0.21; 95% CI, 0.18 to 0.24; *p* < 0.01). SC (overall and individual domains) was associated with functioning, with small-to-medium^a^ ES (ES using û_p_ for overall SC and functioning was 0.24; 95% CI, 0.19 to 0.28; p < 0.01). SC mediated the relationship between NC and functioning (û_p_ = 0.14; p < 0.01). SC and NC together explained 9.2% of the variance in functioning. SC alone explained an additional 4.8% of the variance after accounting for NC; NC alone explained an additional 1.9% of the variance after accounting for SC.
Irani 2012 [Q = High]	SLR with meta-analysis	Total studies: 25 (*n* = 1306)	SCZ spectrum disorders	Only SC (EP)	Domains: social, occupational, independent living (FC and RWF)	There was a significant association between EP and functioning, with medium^a^ ES (δ = 0.31; 95% CI, 0.13 to 0.49; *p* = 0.001).
Schmidt 2011 [Q = Low]	SLR	Total studies: 15 (*n* = 148)	SCZ	Only NC (SC was assessed as mediator)	Domains: social, occupational, independent living (Mostly RWF)	There was a significant indirect relationship between NC and functioning (mean ES of indirect effect, 0.20); SC mediated this relationship.
Mike 2019 [Q = 5/9]	CS	90	SCZ, SAD	NC and SC	Domain: social (Only RWF)	An exploratory factor analysis for SC structure revealed a 3-factor solution representing the domains of emotion recognition, emotion management, and ToM; of these, only ToM was significantly associated with better social functioning.
Rocca 2016 [Q = 7/9]	CS	809	SCZ	NC and SC	Domains: social, occupational independent living (Only RWF)	SCs were organized into 3 clusters - unimpaired, impaired, and very impaired SC. RWF was highest in the unimpaired cluster and lowest in the very impaired cluster. Of the different SC domains, the ToM domains were most important for the cluster definition.
Strassnig 2015 [Q = 6/9]	CS	821	SCZ, SAD	Only NC	Domains: social, occupational independent living (FC and RWF)	For independent living and occupational functioning: correlations were strongest with NC (*r* = 0.10 to 0.16 and *r* = 0.20 to 0.28, respectively) and FC (*r* = 0.29 to 0.42 and 0.21 to 0.30, respectively). For social functioning: correlations were strongest with negative symptoms (r = -0.42 to -0.38).
Shamsi 2011 [Q = 8/9]	CS	185	SCZ, SAD	NC and SC	Domains: social, occupational independent living (Only RWF)	SC was associated only with social functioning (*p* = 0.027). NC domains were associated with occupational functioning (p = -0.44) and independent living (*p* = 0.048), but not with social functioning (attention/vigilance showed only a marginal association with social functioning [*p* = 0.054, not significant]).

CI, confidence interval; CS, cross-sectional study; EP, emotion perception; ES, effect size; FC, functional capacity; NC, neurocognition; Q, quality; r, Pearson's correlation coefficient; RWF, real-world functioning; SAD, schizoaffective disorder; SC, social cognition; SCZ, schizophrenia; SLR, systematic literature review; ToM, theory of mind; û_p_, mean correlation estimate.

a ES interpretation as reported by the respective study authors.

##### 3.1.1.1 Strength of the association between cognition and functioning

In an SLR and meta-analysis covering 12,868 patients with non-affective psychosis across 166 studies (Halverson et al., 2019), both NC and SC were significantly associated with functioning (mean correlation estimate [û_p_] = 0.21; 95% CI, 0.18 to 0.24; *p* < 0.01 for overall NC, i.e. across all NC domains, and û_p_ = 0.24; 95% CI, 0.19 to 0.28; p < 0.01 for overall SC). This association was significant for both RWF and FC domains. SC and NC together explained 9.2% of the variance in functioning. The authors noted that the strength of the association appeared to be stronger for SC than NC; as SC alone explained an additional 4.8% of the variance in functioning after accounting for NC, while NC alone explained only an additional 1.9% of the variance in functioning after accounting for SC.

Association between specific cognitive and functioning domains.

In the SLR described earlier (Halverson et al., 2019), û_p_ across individual NC and functioning domains ranged from 0.06 to 0.33, with the strongest effect seen between overall NC and social skills (û_p_ = 0.33; *p* < 0.001).

Within the individual SC domains, ToM and emotion perception appear to be particularly relevant. An exploratory factor analysis for SC structure revealed a 3-factor solution representing the domains of emotion recognition, emotion management, and ToM; of these, only ToM was significantly associated with functioning (Mike et al., 2019; *n* = 90). A cluster analysis for SC severity yielded 3 clusters (unimpaired, impaired, and very impaired SC) that were associated with RWF; functioning was worst in those with very impaired SC. In this analysis, the ToM domains were the most important for determining the SC clusters (Rocca et al., 2016; *n* = 809). In the SLR by Halverson et al. (2019), û_p_ across individual SC and functioning domains ranged from 0.08 to 0.38, with the strongest effect seen between ToM and social skills (û_p_ = 0.38; *p* < 0.001). An SLR and meta-analysis focusing on emotion perception (Irani et al., 2012) concluded that emotion perception was significantly associated with functioning (RWF and FC; δ = 0.31; 95% CI 0.13 to 0.49; *p* = 0.001).

In terms of specific functioning domains, occupational functioning and independent living were associated with NC, while social functioning was associated with SC. In a large study using confirmatory factor analysis to predict relationships between variables (Strassnig et al., 2015; *n* = 821), NC influenced independent living and vocational functioning; but not social functioning, which was primarily influenced by negative symptoms (the model did not include SC). Broadly similar results were reported by Shamsi et al. (2011; *n* = 185) using logistic regression analyses: SC was associated only with social functioning, while NC domains were associated with work/education and residential status but not with social functioning. In the SLR by Halverson et al. (2019), for the functioning domain of ‘community functioning’ (RWF), NC and SC showed comparably strong associations (NC: û_p_ = 0.20; 95% CI, 0.17 to 0.24; SC: û_p_ = 0.21; 95% CI, 0.15 to 0.26). In contrast, for the social functioning domains of ‘social behavior in the milieu’ (RWF) and ‘social problem solving’ (FC), SC appeared to show stronger association: for ‘social behavior in the milieu’, the effect size was 0.14 (95% CI, 0.06 to 0.22) for NC and 0.31 (95% CI, 0.24 to 0.38) for SC; for ‘social problem solving’, the effect size was 0.26 (95% CI, 0.20 to 0.31) for NC and 0.46 (95% CI, 0.25 to 0.62) for SC.

##### 3.1.1.2 Nature of the association of NC and SC with functioning

Cognition had both direct and indirect effects on functioning. Halverson et al. (2019) demonstrated that SC mediated the relationship of NC with functioning (RWF and FC; û_p_ = 0.14; *p* < 0.01). Similar findings were reported by Schmidt et al. (2011) in a review of 15 studies that focused on this relationship: there was a significant indirect relationship between NC and functioning (mostly RWF), with SC as a mediator (mean effect size = 0.20; effect size ranged from 0.11 to 0.28 across individual studies).

Additionally, the relationship between cognition and functioning is mediated and moderated by other variables, particularly negative symptoms. These are covered in detail in Section 3.1.4.

#### 3.1.2 Association of cognition with change in functioning over time

Table 2 summarizes data from review articles (3 SLRs and 2 narrative reviews) as well as key primary studies (10 longitudinal studies), most of which reported data over 2 to 10 years of follow-up. The published data mostly cover the association of NC with functioning.

**Table 2:** Publications on the association of cognition with change in functioning.

Publication name [Quality]	Type of publication	Sample size/No. of studies (total sample size)	FUP	Population	Cognitive domains assessed	Functioning domains assessed	Key results
Santesteban-Echarri 2017 [Q = High]	SLR with meta-analysis	Total studies: 50 (*n* = 6669)	>12 mo	FEP	Only NC	Domains: social, occupational, independent living (Only RWF)	Most baseline cognitive variables (general cognitive ability, attention, processing speed, verbal fluency, verbal memory and working memory) were significantly associated with functioning over time. Correlation between baseline cognition and follow-up functioning/recovery: • General cognitive ability: *r* = 0.183; 95% CI, 0.074 to 0.287; p < 0.001 • Attention: *r* = 0.216; 95% CI, 0.112 to 0.315; *p* < 0.0001 • Working memory: *r* = 0.171; 95% CI, 0.084 to 0.255; *p* < 0.0001 • Verbal fluency: *r* = 0.167; 95% CI, 0.081 to 0.251; p < 0.0001 • Verbal memory: *r* = 0.145; 95% CI, 0.034 to 0.252; *p* = 0.011 • Executive functioning: *r* = 0.064; 95% CI, −0.031 to 0.157; *p* = 0.188 • Processing speed: *r* = 0.197; 95% CI, 0.098 to 0.292; p < 0.0001 • Nonverbal memory and learning: *r* = 0.119; 95% CI, −0.093 to 0.322; *p* = 0.271 • Visuo-motor skills: *r* = 0.143; 95% CI, −0.045 to 0.321; *p* = 0.136
Allott 2011 [Q = High]	SLR	Total studies: 22 (*n* = 1817)	1-7 y	FEP	Only NC	Domains: social, occupational, independent living (Only RWF)	Most cognitive domains (verbal skills, global/general cognition, reasoning and problem solving, and verbal learning and memory) predicted functioning. In 16 of the 22 studies (73%), at least one cognitive domain predicted RWF. RWF was predicted by verbal or language skills in 36% of the studies, global cognition in 31%, and reasoning and problem solving in 26%.
Christensen, 2007 [Q = Medium]	SLR	Total studies: 21 (*n* = 1411)	25 w (median)	SCZ	Only NC	Domains: social, occupational, independent living (FC and RWF)	Baseline NC influenced subsequent employment, work rehabilitation, work skills and work behavior.
Lysaker 2015 [Q = NA]	Narrative review	Total studies: 5 (NA), of which 2 studies reported data on longitudinal association	6 mo	SCZ	Metacognition	Domains: social, occupational, independent living (FC and RWF)	An association between metacognition and functioning was reported during follow-up.
Green 2004 [Q = NA]	Narrative review	Total studies: 18 (NA)	6 mo-20 y	SCZ	Only NC	Domains: social, occupational, independent living (Only RWF)	Association of cognition with RWF was in the medium to large^a^ ES range across studies (ES for correlations: 0.3 to 0.5).
Lam 2018 [Q = 8/9]	PCS	173	Up to 2 y	UHR for psychosis	Only NC	Domains: social, occupational (Only RWF)	Baseline cognition was associated with UHR non-remission (OR, 1.67; 95% CI, 1.09-2.95; *p* = 0.04). Longitudinal changes in cognition were associated with change in functioning, particularly in the remitters; the differential rate of change in cognition fully accounted for the differential rate of change in functioning between remitters and non-remitters.
Amoretti 2016 [Q = 8/9]	PCS	52	2 y	FEP	Only NC	Domains: social, occupational, independent living (Only RWF)	Baseline cognitive reserve significantly predicted RWF at baseline (Univariate regression analysis - for FAST: R^2^, 0.151; *p* = 0.021, and for GAF: R^2^, 0.103; *p* = 0.060). Baseline cognitive reserve significantly predicted RWF at 2 years (Univariate regression analysis - for FAST: R^2^, 0.134; *p* = 0.033, and for GAF: R^2^, 0.130; *p* = 0.042).
Bergh 2016 [Q = 6/9]	PCS	322	5 y, 10 y	SCZ spectrum disorders	Only NC	Domains: social, occupational, independent living (Only RWF)	Baseline GAF (RWF) predicted 10-year NC in univariate analyses (β, 0.02; 95% CI, 0.01 to 0.03; p < 0.001). Poor academic functioning (RWF) at baseline predicted 10-year global cognition (β, −0.12; 95% CI, −0.18 to −0.06; *p* < 0.001), speed of processing (β, −0.12; 95% CI, −0.18 to −0.06; p = 0.001), and VLM (β, −0.11; 95% CI, −0.17 to −0.05; p < 0.001) in multivariate analysis.
Browne 2016 [Q = 7/9]	PCS	179	2-4 w	SCZ, SAD	NC and SC	Domains: social, occupational, independent living (FC and RWF)	SC was significantly associated with FC and RWF (bivariate correlations between SC and social functioning – for UPSA-B: 0.556, p < 0.001; for SSPA: 0.397, p < 0.001; and for SLOF: 0.334, p < 0.001).
Chang 2016 [Q = 7/9]	PCS	114	1 y	SCZ, SAD	Only NC	Domains: social, occupational, independent living (Only RWF)	NC independently predicted RWF, and explained 5.9% of the variance in RWF.
Simons 2016 [Q = 7/9]	PCS	745	3 y	Psychosis	NC and SC	Domains: social, occupational, independent living (Only RWF)	All NC domains at baseline were significantly associated with social functioning at three years, with the exception of verbal memory retention and response shifting task – higher cognition at baseline predicted better long-term RWF. Baseline SC was not associated with longitudinal social functioning.
Lipskaya-Velikovsky 2015 [Q = 7/9]	PCS	Stage 1: 104 Stage 2: 70	6 m	SCZ	Only NC	Domain: independent living (FC and RWF)	NC, along with other variables, was a significant predictor of ADL and IADL. Prediction of ADL by the severity of negative symptoms, NC, and the number of hospitalizations: 51.2% Prediction of IADL by functional capacity, NC, and the number of hospitalizations: 60.1%
Norman 2015 [Q = 8/9]	PCS	113	5 y	FEP	Only NC	Domains: social, occupational (Only RWF)	NC assessed at one-year post-baseline predicted some, but not all, occupational/vocational outcomes such as being in full-time work or studies, time spent in full-time occupation, and being on disability pension.
Heinrichs 2010 [Q = 7/9]	PCS	127	10 m	SCZ, SAD	Only NC	Domains: occupational, independent living (FC and RWF)	NC, along with demographic and clinical factors accounted for 35%–38% of variance in community independence.
Robinson 2004 [Q = 7/9]	PCS	118	8 y	SCZ, SAD	Only NC	Domains: social, occupational (Only RWF)	Following stabilization of acute psychosis, better cognitive functioning independently predicted adequate social/vocational functioning and recovery.

ADL, activities of daily living; B, unstandardized regression coefficient; β (beta), standardized regression coefficient; CI, confidence interval; ES, effect size; FAST, Functioning Assessment Short Test; FC, functional capacity; FEP, first episode psychosis; GAF, Global Assessment of Functioning scale; IADL, instrumental activities of daily living; mo, months; NA, not applicable; NC, neurocognition; OR, odds ratio; PCS, prospective cohort study; Q, quality; R^2^, coefficient of determination; RWF, real-world functioning; SAD, schizoaffective disorder; SC, social cognition; SCZ, schizophrenia; SLOF, Specific Levels of Functioning scale; SLR, systematic literature review; SSPA, Social Skills Performance Assessment; UHR, ultra-high risk for psychosis; UPSA, University of California San Diego Performance-based Skills Assessment; VLM, visual learning and memory; w, weeks; y, years.

a ES interpretation as reported by the respective study authors.

##### 3.1.2.1 Association between baseline cognition and long-term functioning

In their review of 18 longitudinal studies, Green et al. (2004) concluded that baseline cognition predicted long-term RWF (social, occupational and independent living) with effect sizes ranging from 0.3 to 0.5 for correlations, over 6 months to 20 years of follow-up. Allott et al. (2011) and Santesteban-Echarri et al. (2017) examined this association in patients with early psychosis in large SLRs, and reported that baseline NC domains (i.e., general cognitive ability, attention, processing speed, verbal fluency, verbal memory and working memory) were generally associated with subsequent functioning. In a systematic review of the impact of cognition on work outcomes in schizophrenia, Christensen (2007) concluded that baseline NC influenced subsequent employment, work rehabilitation, work skills and work behavior (RWF and FC); while Lysaker et al. (2015; narrative review) reported an association between metacognition and functioning during follow-up.

Table 2 summarizes data from key individual studies. Across studies, baseline NC and SC predicted functioning at up to 5 years of follow-up. Predictors included NC, intelligence quotient and baseline cognitive reserve, in outpatients (Amoretti et al., 2016; Chang et al., 2016; Heinrichs et al., 2010; Norman et al., 2015) as well as in hospitalized patients (Lipskaya-Velikovsky et al., 2015). Robinson et al. (2004) reported that cognitive performance following stabilization of acute psychosis in a large sample of first episode patients was the best predictor of functional recovery 5 years later. Data on the association of SC with long-term functioning were relatively limited. Browne et al. (2016; *n* = 179) reported that baseline SC was significantly associated with the SSPA (FC) and SLOF (RWF) at both initial and follow-up assessments; however, the follow-up assessments were conducted only 2 to 4 weeks after baseline. In a 3-year follow-up study (Simons et al., 2016; *n* = 745), while most NC domains at baseline predicted social functioning (RWF) at 3 years, baseline SC did not show this association, probably due to the relatively mild SC dysfunction in the study sample.

##### 3.1.2.2 Association between change in cognition and change in functioning

In a prospective cohort study in patients at ultra-high risk for psychosis (Lam et al., 2018; *n* = 173), changes in NC over 2 years were associated with changes in functioning (RWF), particularly in those individuals who remitted, i.e. who no longer met the criteria for ultra-high risk after 2 years. The differential rate of change in NC fully accounted for the differential rate of change in functioning between remitters and non-remitters.

Bergh et al. (2016) took the reverse approach, studying the relationship between cognition and functioning by evaluating factors that predicted long-term cognition in 322 patients with schizophrenia spectrum disorders. Baseline GAF (RWF) predicted 10-year NC in univariate analyses (β = 0.02; 95% CI, 0.01 to 0.03; *p* < 0.001), while premorbid academic functioning predicted 10-year NC in multivariate analyses (association of poor premorbid academic function with global cognitive functioning: β = -0.12; 95% CI, −0.18 to −0.06; p < 0.001).

#### 3.1.3 Effect of cognitive interventions on functioning

Our searches covered all cognitive interventions, including both pharmacological and non-pharmacological approaches. However, as cognitive remediation is the only therapy with relatively consistent efficacy on cognitive impairments in schizophrenia, we have focused on the impact of cognitive remediation on NC, and included some additional data on interventions aimed at SC (Table 3). Since the literature in this area is extensive, we summarize only data from review articles (7 SLRs and 1 narrative review).

**Table 3:** Publications on the effect of cognitive interventions on functioning.

Review name [Quality]	Type of review	No. of studies (total sample size)	Population	Intervention	Functioning outcomes assessed	Key results
Neurocognitive interventions						
Prikken 2019 [Q = Medium]	SLR with meta-analysis	Total studies: 24 (*n* = 1262) Studies assessing functioning: 10 (*n* = 521)	SCZ spectrum disorders	Computerized cognitive drill and practice training (adjunctive psychosocial rehabilitation excluded)	Domains: social, occupational, global (Unclear if both FC and RWF included)	Computerized cognitive drill and practice training (without adjunctive psychosocial rehabilitation) did not have significant effects on functioning (ES, 0.19; 95% CI, -0.01 to 0.39; *p* = 0.07).
Chan 2015 [Q = High]	SLR with meta-analysis	Total studies: 9 (*n* = 740)	SCZ, SAD or bipolar affective disorder	CACR	Domain: occupational (Only RWF)	CACR resulted in significantly improved occupational outcomes including employment rate, total days of work in a year, and total annual earnings: • Employment rates: 41% with CACR vs. 24% without CACR (RD, 20%; 95% CI, 5% to 35%) • Total days of work: 19.5 days longer per year with vs without CACR (95% CI, 2.5 to 36.6 days) • Total annual earnings: US$ 959 per year more with vs without CACR (95% CI, US$285 to US$1634)
Revell 2015 [Q = High]	SLR with meta-analysis	Total studies: 11 (*n* = 615)	FEP	CR	Domains: social, independent living (FC and RWF)	In patients with FEP, CR had a significant impact on social functioning and ADLs (ES [Cohen's d], 0.18; 95% CI, 0.01 to 0.36; *p* < 0.05). CR with adjunctive psychosocial rehabilitation had significantly stronger effects on functioning than CR alone (CR plus rehabilitation: ES, 0.39; 95% CI, 0.12 to 0.66, vs CR alone: ES, 0.03; 95% CI, −0.20 to 0.26; *p* < 0.05).
Wykes 2011 [Q = Medium]	SLR with meta-analysis	Total studies: 40 (*n* = 2104) Studies assessing functioning: 19 (n = 1036)	SCZ	CR	Domains: social, occupational, independent living (FC and RWF)	CR had a significant impact on functioning with small to medium^a^ ES (ES [Cohen's d], 0.42; 95% CI, 0.22 to 0.62). CR with adjunctive psychosocial rehabilitation had significantly stronger effects on functioning than CR alone (CR plus rehabilitation: ES, 0.59; 95% CI, 0.30 to 0.88, vs CR alone: ES, 0.28, 95% CI, −0.02 to 0.58; *p* = 0.02).
McGurk 2007 [Q = Medium]	SLR with meta-analysis	Total studies: 26 (*n* = 1151) Studies assessing functioning: 11 (*n* = 615)	SCZ	CR	Domains: social, occupational, independent living (FC and RWF)	CR had a significant impact on functioning, with small to medium^a^ ES (ES, 0.35; 95% CI, 0.07 to 0.62). CR with adjunctive psychosocial rehabilitation had significantly stronger effects on functioning than CR alone (CR plus rehabilitation: ES, 0.47, vs CR alone: ES, 0.05; *p* < 0.01).
Medalia 2013 [Q = NA]	Narrative review	NA	SCZ	CR	Domains: social, occupational, independent living (FC and RWF)	CR was most likely to impact functional outcome when individuals were given opportunities to practice their cognitive skills in real-world settings. Functioning was enhanced by integrating CR programs with psychosocial rehabilitation programs.

Social cognitive interventions						
Vass 2018 [Q = Medium]	SLR	Total studies: 17 (*n*=681) Studies assessing functioning: 12 (*n* = 490)	SCZ, SAD	SC domain targeted: ToM 5 ToM-focused studies and 7 non-ToM studies	Domains: social, occupational, independent living (FC and RWF)	Both targeted ToM interventions and non-ToM interventions generally showed an effect on social functioning. ToM-focused interventions did not appear to be specifically associated with improved functioning compared to other SC interventions.
Grant 2017 [Q = Medium]	SLR	Total studies: 32 (*n* = 1440) Studies assessing functioning: 19 (NR)	SCZ, SAD	SC domains targeted: ToM, AR, AS and SP	Domains: social, occupational, independent living (FC and RWF)	Limited evidence showed that SC interventions translated into functioning improvement. Specifically, only improved SP appeared to be significantly associated with functioning. Of the 19 studies, 10 reported significant impact of the SC intervention on functioning. Co-occurrence of significant improvement in SC with significant improvement in functioning was seen only for SP (p = 0.02).

ADL, activities of daily living; AR, affect recognition; AS, attributional state; CACR, computer-assisted cognitive remediation; CI, confidence interval; CR, cognitive remediation; ES, effect size; FC, functional capacity; FEP, first episode psychosis; NA, not applicable; NR, not reported; Q, quality; RD, risk difference; RWF, real-world functioning; SAD, schizoaffective disorder; SC, social cognition; SCZ, schizophrenia; SLR, systematic literature review; SP, social perception; ToM, theory of mind; US, United States.

a ES interpretation as reported by the respective study authors.

##### 3.1.3.1 Effect of cognitive remediation on functioning

Overall, cognitive remediation has a clear impact on functioning. In a meta-analysis involving 19 studies reporting data on functioning outcomes (RWF and FC) in patients with schizophrenia (Wykes et al., 2011; *n* = 1036), cognitive remediation was significantly associated with improvement in functioning (RWF and FC; Cohen's d, 0.418; 95% CI, 0.216 to 0.620). In patients with early psychosis, cognitive remediation appeared to have a comparatively small effect on functioning (RWF and FC; Cohen's d, 0.18; 95% CI, 0.01 to 0.36) in studies ranging from 8 weeks to 2 years in duration (Revell et al., 2015). The authors noted that the effect size in their review was smaller than that in reviews involving patients with chronic schizophrenia (McGurk et al., 2007; Wykes et al., 2011), possibly because of the reduced scope of improvement in early disease. They also noted that the study participants analyzed in their review may have been functioning at a higher level at baseline, given that 73% of the studies in their review included only outpatient participants, compared to 50% of the studies in Wykes et al., 2011.

In these reviews assessing the impact of cognitive remediation (McGurk et al., 2007; Revell et al., 2015; Wykes et al., 2011), adding psychosocial rehabilitation/skills training to cognitive remediation improved the outcomes. Wykes et al. (2011) reported that the effects of cognitive remediation were significantly stronger (*p* = 0.02) in studies that provided adjunctive psychosocial rehabilitation to all patients (effect size: 0.59; 95% CI, 0.30 to 0.88) than in studies that examined cognitive remediation alone (effect size: 0.28; 95% CI, –0.02 to 0.58). Medalia and Saperstein (2013) reviewed cognitive remediation and functioning in schizophrenia, and also concluded that functioning outcomes (RWF and FC) were enhanced by integrating a cognitive remediation program with psychosocial rehabilitation programs. In agreement, Prikken et al. (2019) reported that computerized cognitive drill and practice training *without* adjunctive psychosocial rehabilitation had no significant impact on functioning (RWF and FC; effect size: 0.19; 95% CI, -0.01 to 0.39).

In terms of the impact of cognitive remediation on occupational outcomes, Chan et al. (2015) reported that computer-assisted cognitive remediation (CACR) was associated with significantly higher employment rates (41% vs. 24% in those who did not receive CACR), more working days (19.5 more days per year) and higher earnings (US$ 959 more per year).

##### 3.1.3.2 Effect of SC interventions on functioning

Grant et al. (2017) systematically reviewed interventional studies across all SC domains, and found 10 of the 19 studies included showed a significant impact on functioning (RWF and FC). Almost all the studies that reported improved functioning also reported improvement in ToM. The authors conducted an explorative analysis to assess the co-occurrence of significant findings in SC and functioning; only social perception was significantly associated with functional improvements. In an SLR focused on the ToM domain of SC (Vass et al., 2018; 12 studies), SC interventions were generally associated with improved functioning (RWF and FC).

#### 3.1.4 Factors influencing the association of cognition with functioning

The association of cognition with functioning appears to be indirect, with a number of factors mediating (i.e. explaining) this relationship; in addition, there are a number of moderating factors that influence the strength of the association. In Section 3.1.1, the role of SC as a mediating factor was discussed. This section will review other factors, based on data from 8 review articles (6 SLRs and 2 narrative reviews) and 8 primary studies (7 cross-sectional studies and 1 longitudinal; Table 4).

**Table 4:** Publications on factors influencing the association of cognition with functioning.

Publication name [Quality]	Type of publication	Sample size/No. of studies (total sample size)	Relevant factors	Population	Cognitive domains assessed	Functioning domains assessed	Key results
Halverson 2019 [Q = High]	SLR with meta-analysis	Total studies: 166 (*n* = 12,868)	SC	SCZ spectrum disorders	NC and SC	Domains: social, occupational, independent living (FC and RWF)	SC mediated the relationship between NC and functioning. Relationship between NC and functioning including SC as the mediator was significant (û_p_ = 0.14, p < 0.01).
Revell 2015 [Q = High]	SLR with meta-analysis	Total studies: 11 (*n* = 615)	Adjunctive psychosocial rehabilitation	FEP	Only NC (impact of CR)	Domains: social, independent living (FC and RWF)	CR with adjunctive psychosocial rehabilitation had significantly stronger effects on functioning than CR alone (CR plus rehabilitation: ES, 0.39; 95% CI, 0.12 to 0.66, vs CR alone: ES, 0.03; 95% CI, -0.20 to 0.26; p < 0.05).
Fett 2011 [Q = High]	SLR with meta-analysis	Total studies: 52 (*n* = 2692)	Demographic and illness-related variables	SCZ, SAD, non-affective psychosis	NC and SC	Domains: social, occupational, independent living (FC and RWF)	Gender, age, hospitalization status, and disease duration did not significantly influence the relationship between cognition and functioning: • Male gender: β, -0.01 to 0.01; p-value, 0.10 to 0.99 • Age: β, −0.06 to 0.95; *p*-value, 0.09 to 0.95 • Inpatient vs outpatient: β, −0.07 to 0.03; p-value, 0.06 to 0.96 • Illness duration: β, −0.07 to 0.04; p-value, 0.07 to 0.93
Wykes 2011 [Q = Medium]	SLR with meta-analysis	Total studies: 40 (*n* = 2104) Studies assessing functioning: 19 (*n* = 1036)	Adjunctive psychosocial rehabilitation	SCZ	Only NC (impact of CR)	Domains: social, occupational, independent living (FC and RWF)	CR with adjunctive psychosocial rehabilitation had significantly stronger effects on functioning than CR alone (CR plus rehabilitation: ES, 0.59; 95% CI, 0.30 to 0.88, vs CR alone: ES, 0.28, 95% CI, −0.02 to 0.58; p = 0.02).
Ventura 2009 [Q = Medium]	SLR with meta-analysis	Total studies: 73 (*n* = 6519)	Negative symptoms	SCZ	Only NC	Domains: social, occupational, independent living (FC and RWF)	Negative symptoms mediated the relationship between NC and functioning; this mediation effect was seen for all individual NC domains as well as for the composite NC scores.
McGurk 2007 [Q = Medium]	SLR with meta-analysis	Total studies: 26 (*n* = 1151) Studies assessing functioning: 11 (*n* = 615)	Adjunctive psychosocial rehabilitation	SCZ	Only NC (impact of CR)	Domains: social, occupational, independent living (FC and RWF)	CR with adjunctive psychosocial rehabilitation had significantly stronger effects on functioning than CR alone (CR plus rehabilitation: ES, 0.47, vs CR alone: ES, 0.05; p < 0.01).
Lysaker 2015 [Q = NA]	Narrative review	5 studies (3 for mediation effect)	Metacognition	SCZ	NC and SC	Domains: social, occupational, independent living (FC and RWF)	Metacognitive mastery mediated the impact of NC on social relationships and also predicted a significantly higher average job satisfaction in those receiving a CBT.
Medalia 2013 [Q = NA]	Narrative review	NA	Adjunctive psychosocial rehabilitation	SCZ	Only NC (impact of CR)	Domains: social, occupational, independent living (FC and RWF)	CR was most likely to impact functional outcome when individuals were given opportunities to practice their cognitive skills in real-world settings. Functioning was enhanced by integrating CR programs with psychosocial rehabilitation programs.
Chang 2019 [Q = 7/9]	CS	323	Amotivation	FEP	Only NC	Domains: social, occupational, independent living (Only RWF)	In the network analysis, amotivation played a pivotal role. Amotivation showed the highest node strength and relatively high closeness and betweenness indices. It was connected to all of the other psychopathological variables as well as nodes of psychosocial functioning
Harvey 2019a [Q = 7/9]	CS	312	Negative symptoms (reduced emotional experience)	SCZ, SAD	Only SC	Domain: social (FC and RWF)	In patients with lower severity of negative symptoms, SC significantly predicted social functioning. In patients with greater negative symptom severity, FC, and not SC, predicted social functioning.
Jones 2019 [Q = 5/9]	CS	215	RWF instrument	SCZ, SAD	Only SC	Domain: social (Only RWF)	A subset of schizophrenia patients showed extreme overconfidence, and these people demonstrated the poorest SC performance. The impact of confidence on global self-assessments by patients should be considered when evaluating self-reported findings.
Strassnig 2018 [Q = 6/9]	CS	821	Cognitive dysfunction	SCZ	Only SC	Domains: social, occupational, independent living (FC and RWF)	NC was associated with functioning in patients with significant neuropsychological impairment but not in those who were neuropsychologically normal.
Bhagyavathi 2015 [Q = 7/9]	CS	170	SC, negative symptoms	SCZ, SAD	NC and SC	Domain: social (FC and RWF)	In the final model for the association of cognition with functioning, NC influenced functioning through SC and insight, and SC influenced functioning through motivation and negative symptoms.
Moore 2015 [Q = 8/9]	CS	Two samples: Study 1: SCZ, *n* = 435; BD, *n* = 390 Study 2: SCZ, *n* = 205	Cognitive dysfunction	SCZ, BD	Only NC	Domain: independent living (Only FC)	The relationship between cognition and functioning (FC) appeared to follow a quadratic trend. In patients with poorer cognitive performance, the relationship between cognition and functioning was stronger than it was in patients with better cognitive performance.
Galderisi 2014 [Q = 8/9]	CS	921	SC, avolition	SCZ	NC and SC	Domains: social, occupational, independent living (FC and RWF)	Structural equation models were used. NC exhibited the strongest, association with RWF, and this association was completely indirect. SC was a mediator of the association of NC and RWF. Other factors included positive symptoms, disorganization, avolition, availability of disability pension, access to social and family incentives.
Best 2014 [Q = 8/9]	PCS	136	Negative symptoms	SCZ, SAD	Only NC	Domains: social, occupational, independent living (FC and RWF)	Baseline FC predicted RWF at 18 months only for patients with significant positive or undifferentiated symptoms. For patients with negative or depressive symptoms, FC did not predict RWF, suggesting that negative or depressive symptoms may impair RWF recovery in patients with sufficient FC.

β (beta), standardized regression coefficient; BD, bipolar disorder; CBT, cognitive behavior therapy; CI, confidence interval; CR, cognitive remediation; CS, cross-sectional study; ES, effect size; FC, functional capacity; FEP, first episode psychosis; NA, not applicable; NC, neurocognition; PCS, prospective cohort study; Q, quality; RWF, real-world functioning; SAD, schizoaffective disorder; SC, social cognition; SCZ, schizophrenia; SLR, systematic literature review; û_p_, mean correlation estimate.

##### 3.1.4.1 Role of negative symptoms

Negative symptoms have a substantial influence on functioning in schizophrenia – they have a direct effect on functioning, and also appear to mediate and moderate the association of cognition with functioning. In a meta-analysis of 73 studies (Ventura et al., 2009; *n* = 6519) focused on the role of negative symptoms, the total effects of NC (individual NC domains as well as cognitive composite scores) on community functioning and skills assessment were at least partly mediated via negative symptoms.

Bhagyavathi et al. (2015) and Galderisi et al. (2014) explored models examining inter-relationships across variables and reported that cognition had indirect effects on functioning (RWF); with negative symptoms, especially amotivation, mediating this association. The role of amotivation was also highlighted in a network analysis that examined the relationship among 16 variables in patients with first-episode psychosis (Chang et al., 2019; *n* = 323). Amotivation played a pivotal role in the network of variables, showing the highest node strength and relatively high closeness and betweenness indices. Amotivation and diminished expression displayed differential relationships with other variables, supporting the validity of two-factor negative symptom structure.

The moderating role of negative symptoms was examined in a cross-sectional study (Harvey et al., 2019a; *n* = 312) assessing predictors of social functioning. SC accounted for 9% of the variance in interpersonal functioning (RWF) in patients with lower severity of negative symptoms but did not predict functioning in those with more severe negative symptoms. On the other hand, the presence of significant negative symptoms may impair functional recovery (RWF) in patients despite adequate FC (Best et al., 2014; *n* = 136).

##### 3.1.4.2 Severity of cognitive dysfunction

The severity of cognitive dysfunction may moderate the impact of cognition on functioning. Moore et al. (2015; *n* = 640 across 2 samples) observed that the relationship between cognition and functioning (FC) followed a quadratic curve such that relationship between cognition and functioning was stronger in patients with poorer cognitive performance, and weaker in patients with better cognitive performance. This was further demonstrated in a large study (Strassnig et al., 2018; *n* = 821) in which an association between NC and functioning (RWF) was seen in patients who had neuropsychological impairment (interpersonal functioning: r = -0.23; *p* < 0.01 and vocational outcomes: r = -0.22; p < 0.01), but not in those without neuropsychological impairment. Persistent negative symptoms were associated with poor functioning irrespective of their severity.

##### 3.1.4.3 Metacognition

The potential role of metacognition as a mediator was reviewed by Lysaker et al. (2015). In the 3 studies that examined this, metacognitive mastery (defined as “the ability to use knowledge of one's mental states to respond to social and psychological dilemmas and persist at goal-directed behavior”) mediated the impact of NC on the quality and quantity of social relationships and vocational outcomes.

##### 3.1.4.4 Psychosocial rehabilitation

As discussed in Section 3.1.3, cognitive improvements following cognitive remediation are more likely to translate into better functioning when they are accompanied by psychosocial rehabilitation.

##### 3.1.4.5 Other factors

Significant depressive symptoms may impair functional recovery (RWF) in patients despite adequate FC (Best et al., 2014; *n* = 136). Other variables such as gender, age, hospitalization status, first-episode psychosis sample status and disease duration do not appear to significantly influence the relationship between cognition and functioning (Fett et al., 2011; Halverson et al., 2019).

The choice of instrument used for assessing functioning may also influence the findings. Whether the instrument used assesses FC or RWF may impact the effect size, and therefore the likelihood of demonstrating a significant association between cognition and functioning. Note that instruments assessing FC and RWF are not interchangeable, as FC is a measure of ability in ideal circumstances, while RWF is a measure of actual application of the functioning skills in the real world (see Section 3.1.5). Additionally, some patients with schizophrenia demonstrate substantial overconfidence which correlates poorly with performance on cognitive tests, which in turn is minimally associated with self-reports of functioning. Therefore, self-rated functioning measures must be used with caution in schizophrenia (Jones et al., 2020; *n* = 215).

#### 3.1.5 Relationship between FC and RWF

This section summarizes the role of FC in the association of cognition with RWF, focusing on the relationship between FC and RWF. Data are reported from 2 review articles (1 SLR and 1 narrative review) and 6 primary studies (4 cross-sectional and 2 longitudinal studies; Table 5).

**Table 5:** Publications on the relationship of functional capacity (FC) with real-world functioning (RWF).

Publication name [Quality]	Type of publication	Sample size/No. of studies (total sample size)	Population	Cognitive domains assessed	Functioning domains assessed	Key results
Halverson 2019 [Q = High]	SLR with meta-analysis	Total studies: 166 (*n* = 12,868)	SCZ spectrum disorders	NC and SC	Domains: social, occupational, independent living (FC and RWF)	ES for the association of cognition with functioning were generally larger for FC domains than RWF domains. Cognition and FC: The ES (û_p_) was 0.28 (95% CI, 0.22 to 0.33) for ‘social problem solving’ and 0.25 (95% CI, 0.22 to 0.28) for ‘social skills’ Cognition and RWF: The ES (û_p_) was 0.20 (95% CI, 0.17 to 0.24) for ‘community functioning’ and 0.17 (95% CI, 0.10 to 0.25) for ‘social behavior in the milieu’.
Green 2004 [Q = NA]	Narrative review	Total studies: 18 (NA)	SCZ	Only NC	Domains: social, occupational, independent living (Only RWF)	FC refers to the ability to perform certain tasks and is considered to be a proximal measure (i.e. ‘closer’ to cognition), and is therefore more directly sensitive to changes in cognition. Community functioning (RWF) is a more distal construct (i.e. further away from cognition). FC indicates what a person can do, as opposed to RWF which is a measure of what he or she actually does. Good performance on a measure of FC suggests that the person probably could perform that task in the community if they had the appropriate motivation and opportunity.
Harvey 2019a [Q = 7/9]	CS	312	SCZ, SAD	Only SC	Domain: social (FC and RWF)	In patients with lower severity of negative symptoms, SC significantly predicted social functioning. In patients with greater negative symptom severity, FC, and not SC, predicted social functioning.
Harvey 2019b [Q = 7/9]	CS	158	SCZ	Only NC	Domains: social, occupational, independent living (FC and RWF)	Poorer functioning on the VR tasks was associated with poorer cognition and poorer vocational functioning.
Galderisi 2018 [Q = 9/9]	CS	740	SCZ	NC and SC	Domains: social, occupational, independent living (FC and RWF)	In the network analysis, FC and RWF had the most central role in the inter-relationships across multiple variables in schizophrenia. FC bridged cognition with RWF.
Cardenas 2013 [Q = 6/9]	CS^a^	97	SCZ	NR	Domains: social, occupational, independent living (FC and RWF)	When self-efficacy was high, FC was associated with RWF. When self-efficacy was low, FC was not associated with RWF.
Heinrichs 2010 [Q = 7/9]	PCS	127	SCZ, SAD	Only NC	Domains: occupational, independent living (FC and RWF)	Hierarchical regression analyses were conducted to assess the relation of cognition and functioning. Addition of FC to cognition yielded significant increase in validity only for concurrent and not for subsequent RWF.
Harvey 2013 [Q = 7/9]	PCS	195	SCZ, SAD	Only NC	Domain: independent living (Only FC)	The ability to perform cognitively challenging tests, either for NC or FC was statistically unidimensional. The results implicated a single ability factor that was stably related to both neuropsychological and FC test performance; and raised the question of whether cognitive abilities, measured by neuropsychological tests and FC instruments, were tapping a single ability construct.

CI, confidence interval; CS, cross-sectional study; ES, effect size; FC, functional capacity; NA, not applicable; NC, neurocognition; NR, not reported; PCS, prospective cohort study; Q, quality; RWF, real-world functioning; SAD, schizoaffective disorder; SC, social cognition; SCZ, schizophrenia; SLR, systematic literature review; VR, virtual reality.

a Cross-sectional analysis of an RCT.

FC, which refers to the ability to perform certain tasks, is considered to be a proximal measure and is more directly sensitive to changes in cognition. In contrast, RWF refers to actual functioning in the real world and is considered to be a more distal construct (Green et al., 2004). Changes in cognition may take longer to translate into RWF, and a number of factors may interfere with this translation, as discussed in Section 3.1.4. In an SLR (Halverson et al., 2019), the effect sizes for the association of cognition with functioning were generally larger for FC domains than RWF domains: the effect size (û_p_) was 0.28 (95% CI, 0.22 to 0.33) and 0.25 (95% CI, 0.22 to 0.28) for ‘social problem solving’ and ‘social skills’, respectively (both FC); and 0.20 (95% CI, 0.17 to 0.24) and 0.17 (95% CI, 0.10 to 0.25) for ‘community functioning’ and ‘social behavior in the milieu’, respectively (both RWF).

The value of assessing FC in patients with schizophrenia and its role in the association of cognition and RWF has been debated in the literature (Harvey et al., 2013; Heinrichs et al., 2010). More recent studies provide clearer evidence of value. Galderisi et al. (2018) applied a network analysis to data collected on psychopathologic variables, NC, FC, personal resources, and functioning in stable community-dwelling patients with schizophrenia (*n* = 740; 27 variables). The network analysis showed that FC and RWF were the most central and highly interconnected nodes in the network; importantly, FC bridged cognition with RWF, implying that improving the ability to perform tasks relevant to everyday life was critical in improving community functioning. The association of FC with functioning was also demonstrated in a study involving virtual reality (VR) assessment of FC (Harvey et al., 2019b). In this study, poorer performance on the Virtual Reality Functional Capacity Assessment Test (VRFCAT) was associated with poorer cognition, as well as with poorer scores on work skills (r = -0.23; *p* < 0.01). In addition, SC interacts with social competence and negative symptoms to predict social functioning outcomes (Harvey et al., 2019a), much like non-social FC interacts with NC and negative symptoms to predict non-social functioning outcomes.

A further contributor to the relationship between FC and RWF is self-efficacy. Cardenas et al. (2013) reported that in participants with schizophrenia, those with higher self-efficacy manifested a correlation between FC scores and RW outcomes and those who were low in self-efficacy did not. Their interpretation was that participants with higher levels of self-efficacy may be more motivated to attempt real-world tasks, whether or not they can actually accomplish them, and that their ability predicts the RW outcome. Self-efficacy did not predict RWF; just the correlation between FC and RWF.

**Fig. 3:**
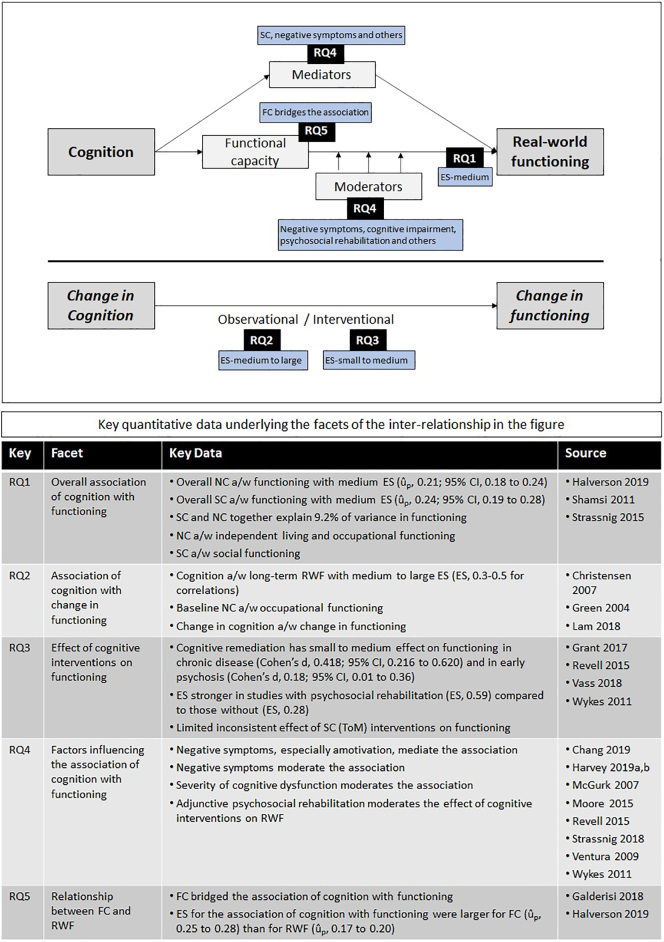
Summary results across all research questions (detailed data presented below the figure). a/w, associated with; ES, effect size; FC, functional capacity; NC, neurocognition; RWF, real-world functioning; RQ, research question; SC, social cognition; ToM, theory of mind.

The results across all research questions are summarized in Fig. 3.

## 4 Discussion

### 4.1 Summary of findings

In this review, we have summarized the relationship of cognition with functioning in schizophrenia, factors that influence this relationship, and the impact of cognitive non-pharmacological interventions. The relationship of NC with functioning has been studied extensively in the literature, including in patients with chronic schizophrenia, first-episode psychosis and at ultra-high risk for psychosis, and in both inpatients and outpatients. NC and SC are significantly associated with functioning (with a medium effect size), and together account for 9.2% of the total variance in functioning, with SC accounting for greater unique variance than NC. Although cognition appears on the surface to explain only a small fraction of the total variance in functioning, it should be noted that the nature of this association is complex and mostly indirect, with many intervening variables. SC itself has been shown to mediate the relationship between NC and functioning. Negative symptoms, especially those related to motivational factors, play a critical role both as a mediator and as a moderator variable - the impact of cognition on functioning is mediated via negative symptoms, and cognition may be able to influence functioning primarily in patients with less severe negative symptoms. This role of negative symptoms as a mediator in the relationship is understandable, since cognitive remediation has been shown to improve negative symptoms including amotivation (Cella et al., 2017 Mar, Cella et al., 2017 Sep 4), and amotivation is known to have a large impact on functional outcomes in schizophrenia (Fervaha et al., 2013). In our review, positive symptoms did not appear to play a substantial role in the relationship of cognition with functioning, which is in keeping with the evidence that positive symptoms have a smaller impact than negative symptoms on functioning in schizophrenia (Rabinowitz et al., 2012). The level of cognitive impairment may moderate the association of cognition with functioning, such that the relationship is stronger in patients with poorer cognitive performance; in patients with better cognitive performance, functioning appears to be primarily influenced by factors such as negative symptoms. Other factors that influence the magnitude of the relationship of cognition with functioning include the presence of depressive symptoms, the use of adjunctive psychosocial rehabilitation, and the instrument selected for assessing functioning. FC bridges the relationship between cognition and RWF, and the effect size for the association of cognition with functioning is generally larger for FC than for RWF, as FC is a proximal construct more directly linked with cognition.

While individual NC domains have empirical validity, these domains are highly correlated with each other and may be best understood as indicators of a global, unifactorial structure. Within individual SC domains, ToM appears to be strongly associated with functioning, suggesting that the ability to understand others' mental states is more directly relevant for functioning in schizophrenia. Overall, SC is more strongly associated with social functioning, while NC is more strongly associated with independent living and occupational functioning, thus highlighting the need for cognitive interventions to be tailored to match the desired functional outcomes.

Cognition also predicts long-term functioning in studies ranging from 6 months to 20 years of follow-up. These studies highlight the economic burden associated with cognitive impairment in schizophrenia, as cognition predicts not only independent living and social functioning, but also occupational functioning in terms of employment, work skills and work behavior.

Several meta-analyses have demonstrated small to medium effects of cognitive remediation on functioning. Cognitive remediation has also been effective in studies involving patients with early psychosis, albeit with smaller effect sizes. Notably, combining cognitive remediation with skills training has resulted in better functional outcomes than cognitive remediation alone. SC-specific data are limited, with some evidence that interventions aimed at ToM and social perception may result in improved functioning. More recently, Nahum et al. (2020) demonstrated improvement in SC and social functioning with the use of an online, plasticity-based training program (SocialVille) in a double-blind RCT involving outpatients with schizophrenia.

### 4.2 Implications for cognitive intervention studies

Given the substantial functional impairment in schizophrenia and the consistent association of cognition with functioning, pharmacological treatments that can improve cognition hold much promise. Based on the findings from this review, we discuss potential implications for studies of pharmacological cognitive interventions in schizophrenia.

Effect sizes are generally comparable between NC and SC, and both are appropriate targets in patients with schizophrenia. Since SC mediates the effect of NC and is therefore more distal in the relationship of cognition with functioning (i.e. it is ‘closer’ to functioning), targeting SC is particularly important.

There is some evidence suggesting that baseline cognitive impairment influences the magnitude of the association between cognition and functioning; this may be investigated further in interventional studies. Similarly, interventional studies may need to consider the potential moderating effect of negative or depressive symptoms on the impact of cognitive interventions on functioning, and minimize/adjust for this effect during recruitment (e.g. by stratification) and statistical analysis.

Based on the data from cognitive remediation studies, it may be beneficial to consider employing adjunctive psychosocial rehabilitation in pharmacological intervention studies to increase the likelihood of translating cognitive improvement into better RWF, although patients in the real world do not always have access to psychosocial rehabilitation. Rehabilitation techniques that focus on negative symptoms may be selected, thereby providing a synergistic effect on functioning (i.e., improved cognition combined with reduced negative symptoms).

Optimal assessment of RWF is a challenge, and will be particularly so in the context of any global study conducted across multiple centers and countries, as the eventual level of community functioning achieved by patients depends on culture- and country-specific factors that cannot be controlled in clinical trials (Buchanan et al., 2005). For assessing RWF, an outcome measure that reports data separately for the functioning domains (independent living, social functioning and occupational functioning) would help in identifying the specific benefits of treatment, especially given the differential impact of NC and SC on these domains. Self-rated RWF scales should be used with caution due to the well-known challenges to self-assessment of everyday outcomes in patients (Silberstein and Harvey, 2019). Researchers could consider employing a measure for FC as the impact of cognitive improvement would be expected to be initially demonstrated on FC, and later translated into RWF.

Finally, any expectation of substantial short- or medium-term improvement in RWF with pharmacological cognitive interventions should be tempered by the modest strength of association between cognition and functioning, and the presence of a number of additional factors that intervene between translation of cognitive improvement into community functioning. Pharmacological interventions might have an increased potential for improving RWF if the interventions are augmented by other training interventions targeting RWF or FC (Harvey and Sand, 2017).

### 4.3 Gaps and limitations

In general, data for SC are limited, especially pertaining to the association of SC with long-term RWF. For non-pharmacological cognitive interventions, robust data are available for cognitive remediation but not for SC interventions, where the literature is limited and inconsistent. Data on the inter-relationships across cognitive and functioning domains are limited, especially for specific RWF domains. Differences across countries and ethnicities have not been addressed in detail in the literature, therefore it is not clear if the findings can be extrapolated across all geographies.

A key limitation of the literature in this field is the interchangeable use of FC and RWF, with reviews often combining data from both types of outcomes. Since effect sizes are generally larger for FC than for RWF, the combined effect sizes reported in the literature may over-state the impact of cognition on RWF.

There is also no consensus on the most appropriate instrument for assessing RWF in schizophrenia, and a wide range of measures have been used. In addition, individual measures have also not always been used consistently, e.g. some studies have reported domain-specific scores on the SLOF, while others have reported only overall functioning scores.

Our review is broad in scope and covers a range of research questions, precluding an in-depth analysis of each aspect of the relationship of cognition with functioning. Nevertheless, we have attempted to cover the most salient findings, and have extracted additional relevant data in the detailed study tables. This is a semi-systematic review and publications were prioritized during selection. While this could have resulted in potentially relevant data being excluded, we have selected the most relevant and high-quality review articles and primary studies, and therefore do not expect that the excluded publications would significantly impact our findings. Since the literature in this area involves review articles with occasional overlap of studies across them, we have been mindful of any such overlap during the selection, analysis and reporting of data from review articles.

## 5 Conclusion

In conclusion, cognition has a modest impact on functioning in schizophrenia and this relationship is indirect and complex. Effective cognitive interventions can alleviate the limitations imposed by cognitive impairment, thereby allowing patients to eventually attain the full potential of community functioning that is currently denied to them.

There is a need for development of models involving cognition and other variables that can together explain a large proportion of the variance in functioning, and which are replicated across different patient populations as well as different cultures. Additional research will also be helpful for understanding how RWF changes with change in SC, including the time period for translating cognitive improvements into better functioning. There is a need for longitudinal studies focused on the association between specific cognitive parameters (NC, SC) and individual RWF domains. Future reviews and meta-analyses may also consider reporting data separately for RWF and FC outcomes.

## Funding source

10.13039/100008349Boehringer Ingelheim (the sponsor) provided financial support for the conduct of the research and preparation of the article. Representatives of the sponsor named as authors contributed to the collection, analysis and interpretation of data; drafting and review of the report; and in the decision to submit the article for publication.

## CRediT authorship contribution statement

Dr. Harvey has received consulting fees or travel reimbursements from Alkermes, Boehringer Ingelheim, Intra Cellular Therapies, Otsuka America, Roche, Sanofi Pharma, Sunovion Pharma, Takeda Pharma, and Teva Pharma. He has a research grant from Takeda and from the Stanley Medical Research Foundation. S Kharawala and H Shukla are employees of Bridge Medical. C Hastedt, J Podhorna, and B Kappelhoff are employees of Boehringer Ingelheim.

## Appendix A Supplementary data

Supplementary tables Image 1

## References

[bb0005] Allott K., Liu P., Proffitt T.-M., Killackey E. (2011). Cognition at illness onset as a predictor of later functional outcome in early psychosis: systematic review and methodological critique. Schizophr. Res..

[bb0010] American Psychiatric Association (2004). Practice guideline for the treatment of patients with schizophrenia, second edition. https://psychiatryonline.org/pb/assets/raw/sitewide/practice_guidelines/guidelines/schizophrenia.pdf.

[bb0015] Amoretti S., Bernardo M., Bonnin C.M. (2016). The impact of cognitive reserve in the outcome of first-episode psychoses: 2-year follow-up study. Eur. Neuropsychopharmacol..

[bb0020] Bergh S., Hjorthøj C., Sørensen H.J. (2016). Predictors and longitudinal course of cognitive functioning in schizophrenia spectrum disorders, 10 years after baseline: the OPUS study. Schizophr. Res..

[bb0025] Best M.W., Gupta M., Bowie C.R., Harvey P.D. (2014). A longitudinal examination of the moderating effects of symptoms on the relationship between functional competence and real world functional performance in schizophrenia. Schizophr. Res. Cogn..

[bb0030] Bhagyavathi H.D., Mehta U.M., Thirthalli J. (2015). Cascading and combined effects of cognitive deficits and residual symptoms on functional outcome in schizophrenia - a path-analytical approach. Psychiatry Res..

[bb0035] Bowie C.R., Harvey P.D. (2006). Cognitive deficits and functional outcome in schizophrenia. Neuropsychiatr. Dis. Treat..

[bb0040] Browne J., Penn D.L., Raykov T. (2016). Social cognition in schizophrenia: factor structure of emotion processing and theory of mind. Psychiatry Res..

[bb0045] Buchanan R.W., Davis M., Goff D. (2005). A summary of the FDA-NIMH-MATRICS workshop on clinical trial design for neurocognitive drugs for schizophrenia. Schizophr. Bull..

[bb0050] Cardenas V., Abel S., Bowie C.R. (2013). When functional capacity and real-world functioning converge: the role of self-efficacy. Schizophr. Bull..

[bb0055] Carrión R.E., Walder D.J., Author A.M. (2018). From the psychosis prodrome to the first-episode of psychosis: no evidence of a cognitive decline. J. Psychiatr. Res..

[bb0060] Cella M., Preti A., Edwards C., Dow T., Wykes T. (2017). Cognitive remediation for negative symptoms of schizophrenia: a network meta-analysis. Clin. Psychol. Rev..

[bb0065] Cella M., Stahl D., Morris S., Keefe R.S.E., Bell M.D., Wykes T. (2017). Effects of cognitive remediation on negative symptoms dimensions: exploring the role of working memory. Psychol. Med..

[bb0070] Chan J.Y.C., Hirai H.W., Tsoi K.K.F. (2015). Can computer-assisted cognitive remediation improve employment and productivity outcomes of patients with severe mental illness? A meta-analysis of prospective controlled trials. J. Psychiatr. Res..

[bb0075] Chang W.C., Hui C.L.M., Chan S.K.W., Lee E.H.M., Chen E.Y.H. (2016). Impact of avolition and cognitive impairment on functional outcome in first-episode schizophrenia-spectrum disorder: a prospective one-year follow-up study. Schizophr. Res..

[bb0080] Chang W.C., Wong C.S.M., Or P.C.F. (2019). Inter-relationships among psychopathology, premorbid adjustment, cognition and psychosocial functioning in first-episode psychosis: a network analysis approach. Psychol. Med..

[bb0085] Christensen T.Ø. (2007). The influence of neurocognitive dysfunctions on work capacity in schizophrenia patients: a systematic review of the literature. Int. J. Psychiatry Clin. Pract..

[bb0090] Czaja S.J., Loewenstein D.A., Lee C.C., Fu S.H., Harvey P.D. (2017). Assessing functional performance using computer-based simulations of everyday activities. Schizophr. Res..

[bb0095] Fervaha G., Foussias G., Agid O., Remington G. (2013 Dec 15). Amotivation and functional outcomes in early schizophrenia. Psychiatry Res..

[bb0100] Fett A.-K.J., Viechtbauer W., Dominguez M.-D.-G., Penn D.L., van Os J., Krabbendam L. (2011). The relationship between neurocognition and social cognition with functional outcomes in schizophrenia: a meta-analysis. Neurosci. Biobehav. Rev..

[bb0105] Fett A.-K.J., Velthorst E., Reichenberg A. (2019). Long-term changes in cognitive functioning in individuals with psychotic disorders: findings from the Suffolk County mental health project. JAMA Psychiatry.

[bb0110] Galderisi S., Rossi A., Rocca P. (2014). The influence of illness-related variables, personal resources and context-related factors on real-life functioning of people with schizophrenia. World Psychiatry.

[bb0115] Galderisi S., Rucci P., Kirkpatrick B. (2018). Interplay among psychopathologic variables, personal resources, context-related factors, and real-life functioning in individuals with schizophrenia a network analysis. JAMA Psychiatry.

[bb0120] Grant N., Lawrence M., Preti A., Wykes T., Cella M. (2017). Social cognition interventions for people with schizophrenia: a systematic review focussing on methodological quality and intervention modality. Clin. Psychol. Rev..

[bb0125] Green M.F., Kern R.S., Heaton R.K. (2004). Longitudinal studies of cognition and functional outcome in schizophrenia: implications for MATRICS. Schizophr. Res..

[bb0130] Green M.F., Llerena K., Kern R.S. (2015). The “right stuff” revisited: what have we learned about the determinants of daily functioning in schizophrenia?. Schizophr. Bull..

[bb0135] Gupta M., Bassett E., Iftene F., Bowie C.R. (2012). Functional outcomes in schizophrenia: understanding the competence-performance discrepancy. J. Psychiatr. Res..

[bb0140] Halverson T.F., Orleans-Pobee M., Merritt C., Sheeran P., Fett A.-K., Penn D.L. (2019). Pathways to functional outcomes in schizophrenia spectrum disorders: meta-analysis of social cognitive and neurocognitive predictors. Neurosci. Biobehav. Rev..

[bb0145] Harvey P.D., Bellack A.S. (2009). Toward a terminology for functional recovery in schizophrenia: is functional remission a viable concept?. Schizophr. Bull..

[bb0150] Harvey P.D., Sand M. (2017). Pharmacological augmentation of psychosocial and remediation training efforts in schizophrenia. Front. Psychiatry..

[bb0155] Harvey P.D., Raykov T., Twamley E.W., Vella L., Heaton R.K., Patterson T.L. (2013). Factor structure of neurocognition and functional capacity in schizophrenia: a multidimensional examination of temporal stability. J. Int. Neuropsychol. Soc..

[bb0160] Harvey P.D., Deckler E., Jarsksog L.F., Penn D.L., Pinkham A.E. (2019). Predictors of social functioning in patients with higher and lower levels of reduced emotional experience: social cognition, social competence, and symptom severity. Schizophr. Res..

[bb0165] Harvey P.D., Khan A., Atkins A., Keefe R.S. (2019). Virtual reality assessment of functional capacity in people with schizophrenia: associations with reduced emotional experience and prediction of functional outcomes. Psychiatry Res..

[bb0170] Heinrichs R.W., Ammari N., Miles A.A., McDermid Vaz S. (2010). Cognitive performance and functional competence as predictors of community independence in schizophrenia. Schizophr. Bull..

[bb0175] Herzog R., Álvarez-Pasquin M.J., Díaz C., Del Barrio J.L., Estrada J.M., Gil Á. (2013). Are healthcare workers' intentions to vaccinate related to their knowledge, beliefs and attitudes? A systematic review. BMC Public Health.

[bb0180] Irani F., Seligman S., Kamath V., Kohler C., Gur R.C. (2012). A meta-analysis of emotion perception and functional outcomes in schizophrenia. Schizophr. Res..

[bb0185] Jones M.T., Deckler E., Laurrari C. (2020). Confidence, performance, and accuracy of self-assessment of social cognition: a comparison of schizophrenia patients and healthy controls. Schizophr. Res. Cogn..

[bb0190] Keefe R.S.E., Davis V.G., Atkins A.S. (2016). Validation of a computerized test of functional capacity. Schizophr. Res..

[bb0195] Lam M., Lee J., Rapisarda A. (2018). Longitudinal cognitive changes in young individuals at ultrahigh risk for psychosis. JAMA Psychiatry.

[bb0200] Lipskaya-Velikovsky L., Kotler M., Easterbrook A., Jarus T. (2015). From hospital admission to independent living: is prediction possible?. Psychiatry Res..

[bb0205] Lysaker P.H., Vohs J., Minor K.S. (2015). Metacognitive deficits in schizophrenia: presence and associations with psychosocial outcomes. J. Nerv. Ment. Dis..

[bb0210] McGurk S.R., Twamley E.W., Sitzer D.I., McHugo G.J., Mueser K.T. (2007). A meta-analysis of cognitive remediation in schizophrenia. Am. J. Psychiatr..

[bb0215] Medalia A., Saperstein A.M. (2013). Does cognitive remediation for schizophrenia improve functional outcomes?. Curr. Opin. Psychiatry..

[bb0220] Mike L., Guimond S., Kelly S. (2019). Social cognition in early course of schizophrenia: exploratory factor analysis. Psychiatry Res..

[bb0225] Moore R.C., Harmell A.L., Harvey P.D. (2015). Improving the understanding of the link between cognition and functional capacity in schizophrenia and bipolar disorder. Schizophr. Res..

[bb0230] Nahum M., Lee H., Fisher M. (2020). Online social cognition training in schizophrenia: a double-blind, randomized, controlled multi-site clinical trial. Schizophr. Bull..

[bb0235] National Institute for Health and Care Excellence (2014). Psychosis and schizophrenia in adults: prevention and management. https://www.nice.org.uk/guidance/cg178/.

[bb0240] Norman R.M.G., Carr J., Manchanda R. (2015). Cognition and the prediction of functioning in patients with a first treated episode of psychosis: a prospective study. Schizophr. Res..

[bb0245] Nuechterlein K.H., Barch D.M., Gold J.M., Goldberg T.E., Green M.F., Heaton R.K. (2004). Identification of separable cognitive factors in schizophrenia. Schizophr. Res..

[bb0250] Pinkham A.E., Penn D.L., Green M.F., Harvey P.D. (2016). Social cognition psychometric evaluation: results of the initial psychometric study. Schizophr. Bull..

[bb0255] Pinkham A.E., Harvey P.D., Penn D.L. (2018). Social cognition psychometric evaluation: results of the final validation study. Schizophr. Bull..

[bb0260] Prikken M., Konings M.J., Lei W.U., Begemann M.J.H., Sommer I.E.C. (2019). The efficacy of computerized cognitive drill and practice training for patients with a schizophrenia-spectrum disorder: a meta-analysis. Schizophr. Res..

[bb0265] Rabinowitz J., Levine S.Z., Garibaldi G., Bugarski-Kirola D., Berardo C.G., Kapur S. (2012 May). Negative symptoms have greater impact on functioning than positive symptoms in schizophrenia: analysis of CATIE data. Schizophr. Res..

[bb0270] Revell E.R., Neill J.C., Harte M., Khan Z., Drake R.J. (2015). A systematic review and meta-analysis of cognitive remediation in early schizophrenia. Schizophr. Res..

[bb0275] Robinson D.G., Woerner M.G., McMeniman M., Mendelowitz A., Bilder R.M. (2004). Symptomatic and functional recovery from a first episode of schizophrenia or schizoaffective disorder. Am. J. Psychiatry.

[bb0280] Rocca P., Galderisi S., Rossi A. (2016). Social cognition in people with schizophrenia: a cluster-analytic approach. Psychol. Med..

[bb0285] Santesteban-Echarri O., Paino M., Rice S. (2017). Predictors of functional recovery in first-episode psychosis: a systematic review and meta-analysis of longitudinal studies. Clin. Psychol. Rev..

[bb0290] Schmidt S.J., Mueller D.R., Roder V. (2011). Social cognition as a mediator variable between neurocognition and functional outcome in schizophrenia: empirical review and new results by structural equation modeling. Schizophr. Bull..

[bb0295] Shamsi S., Lau A., Lencz T. (2011). Cognitive and symptomatic predictors of functional disability in schizophrenia. Schizophr. Res..

[bb0300] Shea B.J., Hamel C., Wells G.A. (2009). AMSTAR is a reliable and valid measurement tool to assess the methodological quality of systematic reviews. J. Clin. Epidemiol..

[bb0305] Silberstein J., Harvey P.D. (2019). Impaired introspective accuracy in schizophrenia: an independent predictor of functional outcomes. Cogn. Neuropsychiatry..

[bb0310] Simons C.J., Bartels-Velthuis A.A., Pijnenborg G.H. (2016). Cognitive performance and long-term social functioning in psychotic disorder: a three-year follow-up study. PLoS One.

[bb0315] Strassnig M.T., Raykov T., O'Gorman C. (2015). Determinants of different aspects of everyday outcome in schizophrenia: the roles of negative symptoms, cognition, and functional capacity. Schizophr. Res..

[bb0320] Strassnig M., Bowie C., Pinkham A.E. (2018). Which levels of cognitive impairments and negative symptoms are related to functional deficits in schizophrenia?. J. Psychiatr. Res..

[bb0325] Vass E., Fekete Z., Simon V., Simon L. (2018). Interventions for the treatment of theory of mind deficits in schizophrenia: systematic literature review. Psychiatry Res..

[bb0330] Ventura J., Hellemann G.S., Thames A.D., Koellner V., Nuechterlein K.H. (2009). Symptoms as mediators of the relationship between neurocognition and functional outcome in schizophrenia: a meta-analysis. Schizophr. Res..

[bb0335] Wells G.A., Shea B., O’Connell D. (2012). The Newcastle-Ottawa Scale (NOS) for assessing the quality of nonrandomised studies in meta-analyses. http://www.ohri.ca/programs/clinical_epidemiology/oxford.asp.

[bb0340] Wykes T., Huddy V., Cellard C., McGurk S.R., Czobor P. (2011). A meta-analysis of cognitive remediation for schizophrenia: methodology and effect sizes. Am. J. Psychiatry.

