# Supplementary material for: The relationship between cognition and functioning in schizophrenia: A semi-systematic review
Source: Schizophr Res Cogn. 2021 Sep 29;27:100217. doi: 10.1016/j.scog.2021.100217 (PMC8488595; doi:10.1016/j.scog.2021.100217)
Supplement: Supplementary file 1 — Supplementary tables [file mmc1.docx]

## Supplementary Information

Supplementary Table S1: Summary protocol

| **Objectives and research questions** | |
| --- | --- |
| Review question(s)/ objective(s) | The objectives of the review were to assess:   - Association of cognition with functioning, including relationships across individual domains - Association of cognition with change in functioning over time - Effect of cognitive interventions on functioning - Factors influencing the association of cognition with functioning - Relationship between functional capacity and real-world functioning outcomes |
| **Studies to include** | |
| Study designs | - Cohort studies (prospective and retrospective) - Cross-sectional studies - Case-control studies - Clinical trials - Review/Systematic review |
| Population | - Population of interest: schizophrenia, schizoaffective disorder, psychosis - Age: no limit - Gender: Any - Race: Any |
| Interventions | - All interventions |
| Publication timeframe | - 2005 - 2019 for full publications   - 2005 – 2019 for systematic searches   - 2000 – 2019 for pragmatic searches - 2013 - 2019 for conference abstracts |
| Language | - No language restriction |
| **Data sources** | |
| Databases | - Embase® and MEDLINE® databases, using Embase.com® search interface |
| Other sources | - Conference proceedings   - American Psychological Association Convention (APA)   - International Society for Pharmacoeconomics and Outcomes Research (ISPOR)   - Schizophrenia International Research Society Conference (SIR) - Bibliographies - Pragmatic searches in PubMed, Google, and Google Scholar - Grey literature searches |
| **Quality assessment** | |
| Quality assessment for risk of bias was conducted using Newcastle-Ottawa Scale for observational studies and AMSTAR for reviews | |

Embase: Excerpta Medica Database; MEDLINE: Medical Literature Analysis and Retrieval System Online

Supplementary Table S2: Search protocol for Embase and MEDLINE databases, conducted using the Embase.com search interface

| **No.** | **Search terms** | **Hits** | **Facet** |
| --- | --- | --- | --- |
| 1 | 'schizophrenia'/exp | 186,911 | Disease |
| 2 | schizophrenia:ab,ti OR CIAS:ab,ti | 145,486 |  |
| 3 | #1 OR #2 | 207,441 |  |
| 4 | 'adl disability'/exp OR 'daily life activity'/exp OR 'lawton instrumental activities of daily living scale'/exp OR 'social interaction'/exp | 146,461 | Functioning |
| 5 | 'functioning':ab,ti OR 'activities of daily living':ab,ti OR adl:ab,ti OR iadl:ab,ti OR 'adl disability':ab,ti OR 'daily life activity':ab,ti OR 'social interaction':ab,ti OR 'social relationship':ab,ti | 282,429 |  |
| 6 | #4 OR #5 | 373,548 |  |
| 7 | 'cognitive defect'/exp OR 'cognitive improvement':ab,ti OR 'cognitive enhancement':ab,ti OR 'cognitive function':ab,ti OR 'cognitive deficits':ab,ti OR 'neurocognitive deficits':ab,ti OR 'executive function':ab,ti OR 'cognitive capacity':ab,ti OR 'cognitive assessment':ab,ti OR 'cognitive performance':ab,ti OR 'cognition':ab,ti | 567,785 | Cognition |
| 8 | #3 AND #6 AND #7 | 5,926 | -- |
| 9 | #3 AND #6 AND #7 AND [conference abstract]/lim | 2,163 | -- |
| 10 | #8 NOT #9 | 3,763 | -- |
| 11 | #8 NOT #9 AND [2005-2019]/py | 3,112 | Final |
